# Participation in community-based healthcare interventions and non-communicable diseases early detection of general population in Indonesia

**DOI:** 10.1016/j.ssmph.2022.101236

**Published:** 2022-09-22

**Authors:** Sujarwoto, Asri Maharani

**Affiliations:** a Department of Public Administration Brawijaya University, Malang, Indonesia; b Department of Nursing, Faculty of Health and Education, Manchester Metropolitan University, UK

**Keywords:** Public health, CBHI, NCDs screening, Indonesia

## Abstract

**Background:**

Community-based Healthcare Interventions (CBHIs) are regarded as a critical component of healthcare task-sharing in LMICs and have the potential to address LMICs’ health system weaknesses to improve NCDs prevention care. This study aims to investigate the relationship between participation in CBHIs and NCDs early detection at medical facilities among Indonesians.

**Methods:**

Data come from the fifth Indonesian Family Life Survey (2014–2015), a total of 27,692 individuals (14,820 female and 12,872 male individuals age 15 and older). Multiple ordered logistics and logistics regression was used to assess the association between individual participation in CBHI and early detection of NCDs at medical facilities.

**Findings:**

Participation in CBHIs are associated with higher odds of having regular blood pressure test (adjusted odds ratio [OR], 3.09; 95% confidence interval [CI], 2.67–3.58), cholesterol test (adjusted OR, 1.88; 95% CI, 1.60–2.22), blood glucose test (adjusted OR, 1.88; 95% CI, 1.58–2.23), electrocardiogram (adjusted OR, 1.37; 95% CI, 1.06–1.76) and basic dental examination (adjusted OR, 1.32; 95% CI, 1.09–1.60) at medical facilities. The odds of having pap smears (adjusted OR, 2.20; 95% CI, 1.62–2.98) and breast self-examination (adjusted OR, 1.73; 95% CI, 1.37–2.19) among females who participated in CBHIs are substantially larger than those who did not participate in CBHIs. No significant association is shown for the basic vision examination (adjusted OR, 1.14; 95% CI, 0.95–1.37), while the association of participation in CBHIs on prostate cancer checkup (adjusted OR, 0.18; 95% CI, 0.04–0.76) was negative and significant. The results were controlled with a wide range of predisposing, enabling and need factors for NCDs early detection.

**Conclusion:**

and recommendation: CBHIs may benefit NCDs early detection for the general population in Indonesia. Policymakers and health practitioners need to design CBHIs programs that are attractive to the population, especially men and younger people.

## Highlights

• CBHIs are a distinct mode of healthcare delivery and are regarded as a critical component of healthcare task-sharing in LMICs.
• CBHIs have the potential to address LMICs' health system weaknesses to improve NCDs prevention care.
• Participation in CBHIs are associated with regular blood pressure, cholesterol, blood glucose, electrocardiogram, and dental tests.

## 1 Introduction

Non-communicable diseases (NCDs) pose a threat to the 2030 Agenda for Sustainable Development in low- and middle-income countries (LMICs), which calls for a one-third reduction in premature deaths from NCDs by 2030 (United Nations, 2015). The World Health Organization's (WHO) Global Status Report highlighted the alarming rise in NCDs in LMICs, particularly cardiovascular diseases (CVDs), diabetes, and cancer. All of these diseases are the biggest burden to LMICs' health systems, especially in the context of universal health coverage. For example, WHO projected that there will be up to 23 million deaths due to CVDs and 21.7 million death in 2030 around the world, with 80% will happen in LMICs (Ferlay et al., 2015), while there will be up to 21.7 million new cancer cases and 13 million cancer-related deaths, with 70% of those occurring in LMICs (Lozano et al., 2012).

As in most other LMICs in the Asia Pacific region, the prevalence of NCDs has risen among the Indonesian population. According to the 2014 Indonesian Sample Registration data, stroke (21.1%), heart disease (12.9%), diabetes mellitus (6.7%), and complications of high blood pressure (5.3%) are the top four most common diseases in the country (Purnamasari, 2019). Based on the Ind onesia Basic Health Research 2018, cancer, stroke, kidney disease, diabetes mellitus, and heart diseases are the major cause of death in 2018 (Purnamasari, 2019). NCDs rates are higher in urban areas than in rural areas in Indonesia. This is because people in urban areas are more likely to engage in risky behaviours such as being sedentary, eating unhealthy foods, and smoking (Purnamasari, 2019).

The reduction in NCDs in high-income countries demonstrates that NCDs can be prevented and controlled with a combination of behavioural, lifestyle, and drug treatment strategies (Bennett et al., 2018). As a result, increasing NCDs awareness, particularly early NCDs screening in LMICs is critical for lowering NCDs rates (Checkley et al., 2014). To curb the rising prevalence of NCDs in the LMICs, WHO further 24 highly cost-effective interventions for NCD, dubbed ‘best buys’, which include tobacco taxation, salt reduction and cervical cancer screening (World Health Organization, 2017). However, reviews of prior studies on LMICs found a lack of evidence for the effectiveness of those interventions (Allen et al., 2017a, Allen et al., 2017b; Allen et al., 2018). WHO further developed a Package of Essential Non-Communicable (PEN) Disease Interventions for primary care in those countries (World Health Organization, 2020). Among the challenges in PEN implementation in LMICs were the lack of health proffesionals and essential medicines (Tripathy & Mishra, 2021). Prior study also revealed that the unmet need for NCDs early detection is common in LMICs due to a lack of health care manpower, limiting the scope of task sharing (Checkley et al., 2014). As highlighted by WHO, people in LMICs frequently lack access to primary health care programmes for early detection and treatment of NCDs' risk factors. People suffering from NCDs in LMICs have less access to effective and equitable health care services that meet their needs. As a result, disease detection is often delayed for many people in these countries, and people die from NCDs at a younger age, often during their most productive years (Checkley et al., 2014).

Accordingly, CBHIs are frequently recognised as a distinct mode of healthcare delivery and are regarded as a critical component of healthcare task-sharing in LMICs (de-Graft Aikins et al., 2014). CBHIs are also seen as a tool for creating healthy community environments in LMICs through broad systemic changes in public policy and community-wide institutions and services (Bennett, 2004). CBHIs’ roles in health care and health in LMIC contexts have been thoroughly documented in studies, but most have focused on communicable diseases, nutrition, and family planning (Abdel-Aziz et al., 2016; Jacobs et al., 2018; Lassi et al., 2014). Nevertheless, previous studies have demonstrated the value of CBHIs in disseminating information about communicable disease prevention as well as providing access to nutrition and contraception services.

Studies on the impact of CBHIs on NCDs in the LMICs contexts yield mixed results. Devkota et al. discovered no link between community-based programmes and hypertension treatment and control in Nepal (Devkota et al., 2016). A large proportion of Nepal's hypertensive population is untreated. Song et al. reported that community-based blood pressure monitoring services had no association with blood pressure control based on cross-sectional data from the 2015 China Health and Retirement Longitudinal Study (CHARLS) (Song et al., 2019). In contrast to these findings, Singh et al. discovered a significant association between CBHIs and NCDs prevention in India (Singh et al., 2011). The findings suggest that the CBHI approach enabled older people to become aware of the risk of NCDs through health education (Singh et al., 2011). Devkota et al. also discovered a link between CBHIs and NCDs prevention in the Cuban population (Devkota et al., 2016). The findings highlight the readiness of health system interventions (i.e., programmes are well equipped and non-health workers are well trained) as the key factors. The mixed results from the literature above indicate that a better understanding of the relationship between CBHIs and hypertension awareness, treatment, and control in various LMIC contexts is needed.

The case of CBHIs in Indonesia could be unique. The Indonesian healthcare system has been characterised by widespread decentralisation, which has given local communities and governments the authority and responsibility to provide healthcare services (Thabrany, 2006). The establishment of various CBHIs is an example of decentralisation in health care. Such community involvement has been critical to the success of various government-mandated programmes, with village health posts or *Posyandu* representing achievements in immunisation, family planning, and nutrition improvement program (Kusuma, 2022). Following the success of the *Posyandu* family planning programme, the Indonesian government established two CBHIs for NCDs in 2011: integrated health posts for NCDs (*Posbindu PTM*) and integrated health posts for older people (*Posyandu Lansia*) (Sujarwoto & Maharani, 2020). *Posbindu PTM* was created specifically for NCD monitoring and counselling in communities; they target people aged 15 and up, whereas *Posyandu Lansia* is concerned with the health of the older people (65 years and older). *Posbindu PTM* personnel consist of 5–8 Kaders (trained community health volunteers) who are trained and supervised by primary healthcare nurses and medical doctors. The main activities in the *Posbindu PTM* and *Posbindu Lansia* include: (1) screening for NCDs, mainly hypertension and diabetes; (2) assessing risk factors, i.e., smoking behaviour, diet, and physical activities; (3) health education; and (4) facilitates referral to primary health care (Indonesia Ministry of Health, 2012; Widyaningsih et al., 2022).

*Kaders* must first complete a three-day training course led by trained nurses and physicians appointed by the Ministry of Health. The Ministry of Health provides the *Posbindu* handbook of technical guidelines, including curriculum and training materials. Kaders' role and health promotion skills, anthropometry, blood pressure measurement, blood glucose and cholesterol measurement, breast self-examination, and some basic knowledge about NCDs diseases are all part of the *Posbindu* technical guidelines. Kaders should achieve a score of more than 80% to pass the training evaluation tests. District governments provide modest funding of approximately IDR 250K–500K (USD 18–36) through Primary Health Care Centres (*Puskesmas*) and community village funds. The funds will cover the costs of Kaders' transportation during health promotion and screenings (Indonesia Ministry of Health, 2012). In 2018, there were 33,679 *Posbindu* PTM in Indonesia. The government of Indonesia has set a target of 136,000 CBHIs for NCDs by 2022. In 2018, early detection of NCDs was at approximately 42 per cent of the target population, compared to the government target of 100 per cent (Indonesia Ministry of Health, 2012). However, few analyses of the potential benefits of CBHIs in NCDs, especially CVDs in Indonesia, have been conducted since the program's inception in 2011.

Thus, this study aims to investigate the relationship between participation in CBHIs for NCDs early detection at medical facilities among Indonesians. We hypothesised that individuals who participate in CBHIs have higher odds of having NCDs early detection at medical facilities than those who do not participate in CBHIs.

## 2 Methods

### 2.1 Study population

The Indonesian Family Life Survey (IFLS) is a longitudinal household survey in Indonesia that collects data through questionnaires as well as physical and laboratory examinations. Individual, household, and community data were collected. The first IFLS (IFLS1) employed a stratified sampling strategy based on province and urban/rural location. 14 of the 27 provinces that existed at the time IFLS1 was conducted were excluded for cost-effectiveness. The sample included 13 of Indonesia's 27 provinces, accounting for 83 per cent of the population: four Sumatra provinces (North Sumatra, West Sumatra, South Sumatra, and Lampung), all five Javanese provinces (DKI Jakarta, West Java, Central Java, DI Yogyakarta, and East Java), and four provinces covering the remaining major island groups (Bali, West Nusa Tenggara, South Kalimantan, and South Sulawesi) (Strauss et al., 2016).

Enumeration areas (EAs) were chosen at random within each province from a nationally representative sample frame used in a socio-economic survey of approximately 60,000 households in 1993. Households were chosen at random within a specific EA. Interviews were conducted with the household heads, their spouse, and up to four randomly selected other household members because interviewing all household members would have been prohibitively expensive. All original household members were tracked throughout four IFLS waves (Strauss et al., 2016).

**Fig. 1:**
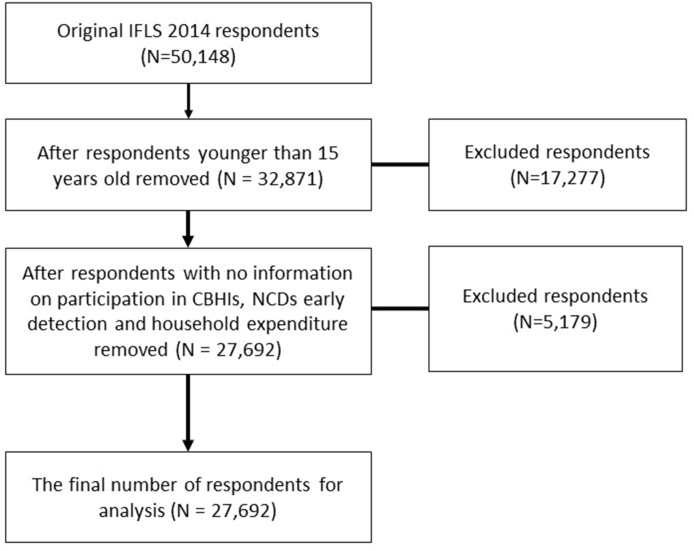
Original number of IFLS 2014, excluded individuals, and final number of respondents included for the analysis.

The current study was based on the fifth wave of the IFLS (IFLS5), which took place in 2014–2015. The IFLS5 tracked both original and split-off households, resulting in a 76 per cent re-contact rate (including death) for original IFLS1 household members and an 82 per cent re-contact rate (including death) for IFLS1 main respondents (Strauss et al., 2016). From the IFLS5, we included 27,692 respondents (12,872 males and 14,820 females) age 15 and older who completed answered questions related to participation in CBHI, NCDs early detection, and household expenditure (Fig. 1).

### 2.2 Main variables measured

The dependent variable in this study is NCDs early detection which consists of (1) blood pressure test; (2) cholesterol test; (3) blood glucose test; (4) electrocardiogram; (5) basic vision examination; (6) basic dental examination; (7) prostate cancer test; (8) pap smear; and (9) breast self-examination. For blood pressure test, cholesterol test, blood glucose test, electrocardiogram examination, basic vision examination, basic dental examination, prostate cancer test, each respondent was asked “when did she/he have last checkup in medical facilities, which include public hospital (general or specialty), public health centre, private hospital, polyclinic, private clinic, medical centre, private physician, nurse, paramedic, or midwife?” If the respondents answer yes, then enumerator will ask “how regularly she/he conducted the checkup?” we coded the answer into three: 0 = never, 1 = irregular, and 2 = regular.

The survey has slightly different questions to answer on pap smears and breast self-exam. For these three questions, the respondent was asked, “How many times did you have a pap smear or perform a breast self-exam in the last 12 months?” We coded the respondent answer into two = 0 = never, 1 = ever”.

The main independent variable in this study was participation in CBHIs. In the survey, respondents were asked with a question “Do you know whether, in the last 12 months, the *Posbindu PTM* or *Posbindu Lansia* activity has occurred in this village?” If they answered “Yes”, the respondents were asked “During the last 12 months, did you participate in or use *Posbindu PTM* or *Posbindu Lansia* in your community?” We categorised respondents as participating in a CBHI for NCDs if they answered “yes” to the interview question.

### 2.3 Control variables

The Anderson model of healthcare-use behaviour was utilised to explore potential determinants of NCD screening (Anderson et al., 2008). This model covers three domains: predisposing, enabling, and need variables, which interact to determine one's health-related behaviour. Predisposing factors include age, sex, marriage status, ethnicity, religion, rural/urban, socio-economic status, lifestyle, and personality. Ethnicity and religion were divided into Javanese and Muslim, who make up the majority of Indonesians, and non-Javanese and non-Muslim. Education was classified into no schooling (as reference variable), elementary, junior secondary, high school, and university. Monthly household expenditure is used as a proxy of household income. Lifestyle was measured using smoking status (currently smoking and never smoking) and physical activity (sedentary lifestyle and non-sedentary lifestyle). Sedentary people were those who did not engage in any moderate or strenuous physical activity and walked less than three times a week (Ford, 2002). The Big Five Inventory (BFI-15) was used to assess personality traits, with scores ranging from 1 to 5 for openness, conscientiousness, extroversion, agreeableness, and neuroticism (Gosling et al., 2003).

Enabling include access to healthcare and insurance coverage. Access to healthcare was measured by the respondent's travel time to the nearest healthcare facilities (<10 min and ≥10 min). Self-reported health insurance ownership was used to measure insurance coverage (1 = covered by any health insurance, 0 = do not have health insurance). Need factors are a parental history of NCDs, metabolic health, mental health, history of comorbidities, and reproductive risk factors. Menopausal status, age at menarche, comorbidities, parental history of cancer death, and body mass index (BMI) computed from measured weight and height during the physical examination were among the criteria reflecting NCDs screening needs. Comorbidities were evaluated using a comorbidity score similar to the Charlson comorbidity index, in which each available co-morbid condition (hypertension, diabetes, asthma, heart disease, liver disease, stroke, cancer, arthritis, kidney disease, stomach or digestive disease, and memory-related disease) contributed one point to the composite index, with additional points awarded for older age (Strauss et al., 2016). Finally, depression was evaluated using a short version (10-item) of the Center for Epidemiologic Studies Depression Scale (CES-D), and a cut-off of 8 was adopted for screening for depressive symptoms (Andresen et al., 2013). All variables, questions, and coding structures are detailed in the **Supplementary File 1 (S1)**.

### 2.4 Statistical analyses

The statistical analysis was conducted in the following steps: Firstly, descriptive statistics were used to describe the study variables in the study population. Secondly, we performed ordered logistic regressions to examine the linkage between participation in CBHIs and NCDs detection (i.e., blood pressure test, cholesterol test, blood glucose test, electrocardiogram exam, basic eye and vision exam, basic dental exam, and prostate cancer test) as the outcome variable was ordinal scale form. Logistic regressions were performed for a pap smear and breast self-exam as both variables were nominal scale form. All models were adjusted with age, sex, marriage status, ethnicity, education, religion, rural/urban, socio-economic status, lifestyle (smoking status and physical activity), and personality. We presented both outcomes using Odd Ratio (OR) and 95% confidence intervals (95% CIs). Survey weight was used in all analyses to adjust for non-response bias. We used the IFLS5 cross-sectional analysis of individual weights for the sampling procedures (which oversampled urban areas and some outer provinces) and for attrition (Strauss et al., 2016). For all statistical analyses, a two-sided *p*-value of <0.05 was conducted. The maximum likelihood (ML) estimator was used to estimate all models; for the probability model, we reported the odds ratio (OR) (Menard, 2002). We used the *svy* command in STATA to include the sampling weights in the analysis. Robustness checks were conducted by performing firth logistic regression due to the small proportion of CBHIs participation in the sample. STATA 17 was used to estimate the models.

## 3 Results

### 3.1 Sample characteristics

Table 1 describes respondents’ participation in CBHIs and characteristics of predisposing, enabling and need factors of NCDs early detection in the survey. The number of respondents participating in CBHIs during the last 12 months was 886 individuals (3.2%). The participation in CBHIs among females (4.5%) was higher than among males.

**Table 1:** Participation in CBHI and other predisposing, enabling and need factors of NCDs early detection.

		Male (N = 12872)	Female (N = 14820)	Total (N = 27692)
		N (% or mean)	N (% or mean)	N (% or mean)
Participate in CBHI	No	12650 (98.3)	14156 (95.5)	26806 (96.8)
	Yes	222 (1.7)	664 (4.5)	886 (3.2)
Predisposing factors				
Age group	15–30	4100 (31.9)	5204 (35.1)	9304 (33.6)
	31–45	5001 (38.9)	5393 (36.4)	10394 (37.5)
	46–65	3091 (24.0)	3468 (23.4)	6559 (23.7)
	65–99	680 (5.3)	755 (5.1)	1435 (5.2)
Marriage status	Unmarried	2580 (20.0)	1699 (11.5)	4279 (15.5)
	Married	9810 (76.2)	11332 (76.5)	21142 (76.3)
	Separated	42 (0.3)	91 (0.6)	133 (0.5)
	Divorced	201 (1.6)	407 (2.7)	608 (2.2)
	Widowed	236 (1.8)	1287 (8.7)	1523 (5.5)
	Cohabitate	3 (0.0)	4 (0.0)	7 (0.0)
Education	No schooling	518 (4.0)	1091 (7.4)	1609 (5.8)
	Elementary	4183 (32.5)	5089 (34.3)	9272 (33.5)
	JSE	2134 (16.6)	2539 (17.1)	4673 (16.9)
	High school	4425 (34.4)	4275 (28.8)	8700 (31.4)
	University	1612 (12.5)	1826 (12.3)	3438 (12.4)
Monthly household expenditure (IDR)		(Mean) 1.14 million (SD = 1.05 million)	(Mean) 1.09 million (SD = 946.725)	(Mean) 1.11 million (SD = 998408)
Religion	Non-Muslim	1291 (10.0)	1415 (9.5)	2706 (9.8)
	Muslim	11581 (90.0)	13405 (90.5)	24986 (90.2)
Ethnicity	Java	7347 (57.1)	8635 (58.3)	15982 (57.7)
	Non-Java	5525 (42.9)	6185 (41.7)	11710 (42.3)
Resident living	Rural	5382 (41.8)	6147 (41.5)	11529 (41.6)
	Urban	7490 (58.2)	8673 (58.5)	16163 (58.4)
Smoking status	No	3100 (24.1)	14320 (96.6)	17420 (62.9)
	Yes	9772 (75.9)	500 (3.4)	10272 (37.1)
Physical activity	Sedentary	1026 (14.4)	1347 (18.0)	2373 (16.3)
	Lightly activity	1061 (14.9)	953 (12.8)	2014 (13.8)
	Moderate activity	2010 (28.3)	4280 (57.3)	6290 (43.1)
	Vigorous activity	3017 (42.4)	885 (11.9)	3902 (26.8)
Openness	<4	2015 (15.7)	3110 (21.0)	5125 (18.5)
	≥4	10857 (84.3)	11710 (79.0)	22567 (81.5)
Conscientiousness	<4	1151 (8.9)	1513 (10.2)	2664 (9.6)
	≥4	11721 (91.1)	13307 (89.8)	25028 (90.4)
Extroversion	<4	4899 (38.1)	4523 (30.5)	9422 (34.0)
	≥4	7973 (61.9)	10297 (69.5)	18270 (66.0)
Agreeableness	<4	694 (5.4)	877 (5.9)	1571 (5.7)
	≥4	12178 (94.6)	13943 (94.1)	26121 (94.3)
Neuroticism	<4	9909 (77.0)	9819 (66.3)	19728 (71.2)
	≥4	2963 (23.0)	5001 (33.7)	7964 (28.8)
*Enabling factors*				
Travel time	<10 min	11985 (93.1)	13039 (88.0)	25024 (90.4)
	≥10 min	887 (6.9)	1781 (12.0)	2668 (9.6)
Health insurance ownership	No	11033 (85.7)	12585 (84.9)	23618 (85.3)
	Yes	1839 (14.3)	2235 (15.1)	4074 (14.7)
*Need factors*				
Parent died from CVD	No	11996 (93.2)	13815 (93.2)	25811 (93.2)
	Yes	876 (6.8)	1005 (6.8)	1881 (6.8)
Parent died from diabetes	No	12670 (98.4)	14559 (98.2)	27229 (98.3)
	Yes	202 (1.6)	261 (1.8)	463 (1.7)
Parent died from cancer	No	12723 (98.8)	14644 (98.8)	27367 (98.8)
	Yes	149 (1.2)	176 (1.2)	325 (1.2)
Number of comorbidities	<1	12350 (95.9)	13926 (94.0)	26276 (94.9)
	≥2	522 (4.1)	894 (6.0)	1416 (5.1)
CESD	<8	9366 (72.8)	10604 (71.6)	19970 (72.1)
	≥8	3506 (27.2)	4216 (28.4)	7722 (27.9)
BMI	<25 kg/m2	9903 (76.9)	8786 (59.3)	18689 (67.5)
	≥25 kg/m2	2969 (23.1)	6034 (40.7)	9003 (32.5)
Menopausal status	Premenopausal		9704 (85.8)	
	Postmenopausal		1606 (14.2)	
Age at menarche	<14		10621 (93.9)	
	≥14		689 (6.1)	

The mean age of the respondents was 39.64 ± 18.66 years, while the mean age of the female respondent was slightly older at 39.71 ± 18.89 years. Most respondents were 31–45 years old (37.5%). The majority of respondents were married (76.3%). About one-third (33.5%) of all respondents are educated from elementary school. Male respondents have higher education than female respondents. The average monthly household expenditure was 1,14 million rupiahs (or about 80US$). Female households have lower monthly household expenditures than male households. Most respondents are Muslim and have Javanese ethnic background (90.2% and 57.7%).

More than half of the respondents live in urban areas (58.4%). Among 27,692 respondents, 37.1% reported smoking, with the majority of smokers being male respondents (75.9% male reported smoking). Most of the respondents reported having moderate physical activities (43.1%). Among female respondents, 57.3% reported having moderate physical activities, while among male respondents, 42.2% reported having vigorous physical activity. Most respondents reported visiting the nearest health facilities in less than 10 min (90.4%). Only about 14–15% of respondents were covered by health insurance. About 6.8%, 2% and 1% of respondents’ parents died from CVDs, diabetes, and cancer, respectively. Approximately 6–7% of respondents reported having two or more comorbidities. Nearly a quarter (27.9%) of respondents have mental health issues. The proportion of overweight respondents (BMI ≥25 kg/m2) was 32.52%, while that of females was 40.7%. Also, 14.2% of females were postmenopausal, while 6.1% reported menarche at 14 and older.

### 3.2 NCDs early detection profile

Table 2 describes NCDs early detection profile among 27,692 respondents in the survey. Overall, NCDs early detection are very low within the population. Only 3996 (14.4%) of respondents reported having regular blood pressure checkups. Less than two per cent or respondents reported having regular cholesterol, blood glucose, electrocardiogram and prostate cancer tests. About one per cent of respondents reported having regular basic vision and dental examinations. Only about 4% of females reported having pap smears, while 15.3% of females reported ever conducting breast self-examinations.

**Table 2:** NCDs early detection profile.

Variable		Male (N = 12872)	Female (N = 14820)	Total (N = 27692)
Blood pressure test	Never	2963 (23.0%)	1541 (10.4%)	4504 (16.3%)
	Irregular	9258 (71.9%)	9934 (67.0%)	19192 (69.3%)
	Regular	651 (5.1%)	3345 (22.6%)	3996 (14.4%)
Cholesterol test	Never	11293 (87.7%)	12820 (86.5%)	24113 (87.1%)
	Irregular	1357 (10.5%)	1813 (12.2%)	3170 (11.4%)
	Regular	222 (1.7%)	187 (1.3%)	409 (1.5%)
Blood glucose test	Never	11389 (88.5%)	13109 (88.5%)	24498 (88.5%)
	Irregular	1230 (9.6%)	1475 (10.0%)	2705 (9.8%)
	Regular	253 (2.0%)	236 (1.6%)	489 (1.8%)
Electrocardiogram	Never	12090 (93.9%)	14163 (95.6%)	26253 (94.8%)
	Irregular	640 (5.0%)	603 (4.1%)	1243 (4.5%)
	Regular	142 (1.1%)	54 (0.4%)	196 (0.7%)
Basic eye and vision exam	Never	11065 (86.0%)	12459 (84.1%)	23524 (84.9%)
	Irregular	1620 (12.6%)	2218 (15.0%)	3838 (13.9%)
	Regular	187 (1.5%)	143 (1.0%)	330 (1.2%)
Basic dental exam	Never	11172 (86.8%)	12713 (85.8%)	23885 (86.3%)
	Irregular	1538 (11.9%)	1913 (12.9%)	3451 (12.5%)
	Regular	162 (1.3%)	194 (1.3%)	356 (1.3%)
Prostate cancer test	Never	12600 (97.9%)		
	Irregular	199 (1.5%)		
	Regular	73 (0.6%)		
Pap smear	Never		14196 (95.8%)	
	Ever		624 (4.2%)	
Breast self-exam	Never		12546 (84.7%)	
	Ever		2274 (15.3%)	

### 3.3 Bivariate and multiple regression analyses

Table 3 shows the bivariate associations between participation in CBHIs and each variable of NCDs early detection. Participation in CBHIs was significantly associated with all NCDs early detection. A negative association was found for prostate cancer checkup. Next, we included predisposing, enabling and need factors of NCDs early detection in the model, starting with early detection for CVDs (blood pressure, blood glucose, cholesterol test and electrocardiogram). The detailed results of multiple regression analyses are presented in **Supplementary File 2 (S2)**.

**Table 3:** Bivariate correlation between participation in CBHI and awareness of NCDs early detection.

Variable	OR.	p-value
Blood pressure test	4.17	0.000
Cholesterol test	3.21	0.000
Blood glucose test	3.12	0.000
Electrocardiogram	2.03	0.000
Basic eye and vision exam	1.57	0.000
Basic dental exam	1.30	0.002
Prostate cancer test	0.53	0.277
Pap smears	3.19	0.000
Breast self-examination	1.25	0.019

**Fig. 2:**
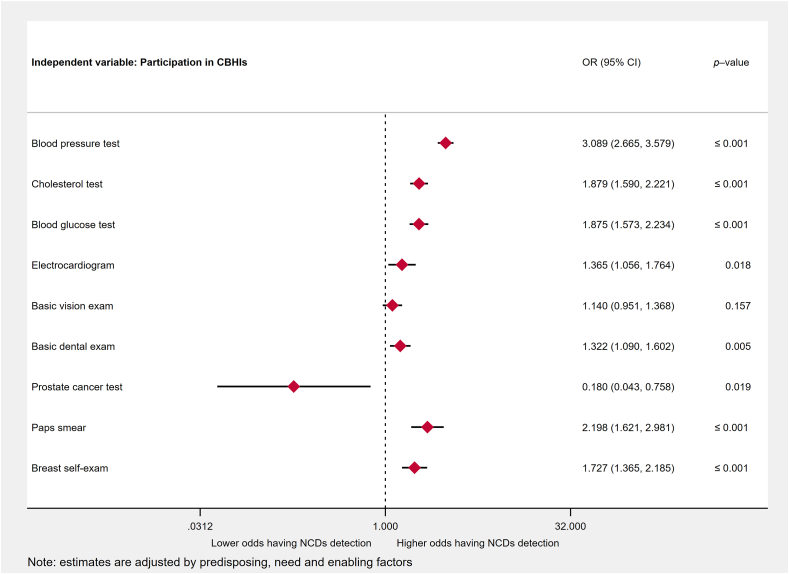
Multiple ordered logistic regression results of participation in CBHI and early CVDs detection.

Fig. 2 shows multiple ordered logistic regression results of participation in CBHI and early CVDs detection. After including predisposing, enabling and need factors, the associations of participation in CBHIs and the odds of having a blood pressure test (adjusted odds ratio [OR], 3.09; 95% confidence interval [CI], 2.67–3.58), cholesterol test (adjusted OR, 1.88; 95% CI, 1.60–2.22), blood glucose test (adjusted OR, 1.88; 95% CI, 1.58–2.23), and electrocardiogram (adjusted OR, 1.37; 95% CI, 1.06–1.76) remain positive and significant. However, the association of CBHIs with basic vision examination diminished (adjusted OR, 1.14; 95% CI, 0.95–1.37). The association of CBHIs with basic dental examination remains significant (adjusted OR, 1.32, 95% CI, 1.09–1.60). The relationship between CBHIs and prostate cancer tests is negative and significant (adjusted OR, 0.18; 95% CI, 0.04–0.76). In contrast, the association between participation in CBHIs and pap smear test (adjusted OR, 2.20; 95% CI, 1.62–2.98) and breast self-exam (adjusted OR, 1.73; 95% CI, 1.37–2.19) are positive and significant.

### 3.4 Predisposing, enabling, and need variables of NCDs early detection

Education and household economic status are consistent predictors of NCDs early detection. Individuals from higher education and economic status have more regular tests than those from a lower education and economic status level. The findings are aligned with the positive association of urban areas with early detection services. As health facilities for medical checkup are mostly available in the urban area where most educated and higher economic status individuals live, they who live in urban areas are likely to have more regular blood glucose, cholesterol, electrocardiogram, eye, dental and cancer early detection checkup.

Healthcare and insurance coverage are significantly associated with CVDs, vision and dental checkups. Individuals who have easier access to health facilities and are covered by health insurance have more regular checkups than those who have difficulties accessing healthcare and are not covered by health insurance. Travel time to the nearest healthcare facilities is associated with female respondents' prostate cancer and pap smear tests, but it is not associated with female respondents’ breast self-exam. The null association of breast self-exam may indicate that the practice is usually conducted at home rather than in health facilities.

Comorbidities and BMI are the consistent predictors of NCDs early detection. Parental history of CVDs and diabetes are associated with more regular blood glucose tests. However, the linkage between parental history of NCDs and early detection is mixed. Smoking status is also a consistent predictor of CVDs early detection, with individuals who reported smoking being less regular to have early detection than those who reported not smoking. However, smokers are less likely to have regular eye checkups than non-smokers. No significant association is shown between smoking status and early detection of cancer.

The older age group is likely to have regular checkups. Accordingly, the relationships of gender, marriage status, religion, ethnicity, sedentary lifestyle, and personality on NCDs early detection are mixed.

### 3.5 Robustness checks

Because the proportion of respondent's participation in CBHIs was small (3.2%), the logistic estimation results may have substantial bias. As King and Zeng (2001) suggested, the firth method or penalised likelihood can be used to reduce small sample bias in maximum likelihood estimation. We performed penalised maximum likelihood logistic regression using *firtlogit* command in STATA. The estimated results of the firth method were relatively similar to the logistic regression presented. The results of firth logistic regressions are presented in **Supplementary File 3 (S3)**.

## 4 Discussion

Based on the nationally representative data, this study investigates the association between participation in CBHIs and early detection of NCDs in a developing country. We found that individuals who participated in CBHIs have higher odds of having NCDs screenings, including blood pressure, cholesterol, blood glucose, ECG, and basic dental examinations. Our findings are in line with prior studies in high-income and LMICs countries that show CBHIs to benefit participants NCD's prevention (Fornari et al., 2013; Mc Namara et al., 2020; Sarrafzadegan et al., 2009). For example, a study in Iran demonstrated that the Isfahan Healthy Heart Program participants improved dietary habits and physical activities after four years (Sarrafzadegan et al., 2009). A community-based study in Brazil further found that a multidisciplinary educational program for children could reduce their parents' cardiovascular risks as measured by the Framingham cardiovascular risk scores after one year (Fornari et al., 2013). A review on CBHIs highlighted the paucity of CBHIs studies from the LMICs, which may be due to overburdened and resource limitations in those countries (Philip et al., 2018). The increasing trend of morbidity and mortality due to NCDs lead to national efforts to prevent and control the diseases. In the last decade, the Government of Indonesia has focused on seven strategic areas to prevent NCDs, including regulations; surveillance; early detection of risk factors; information, education and communication; improvement of case management; improvement of community participation and implementation of NCD programs (Indonesia Ministry of Health, 2017). One of those programmes is providing health promotion and screening activities for NCD risk factors, including blood pressure, blood glucose, and cholesterol tests, as the main activities of Posbindu PTM and Posyandu Lansia (Indonesia Ministry of Health, 2012).

CBHIs may improve the health of the communities using social ecology in which individual behaviour changes due to social influences, including the interventions in CBHIs' program (Golden & Earp, 2012; McLeroy et al., 2003). Participating in CBHIs may improve health-seeking behaviour through improved individual and community knowledge (Abeid et al., 2015). The interaction between individuals and environmental characteristics affecting health outcomes has long been recommended to guide public health practice. The findings in this study could provide an evaluation of those programs on the NCDs’ screening in Indonesia. A review of cardiovascular risk and hypertension-related community programs supported our findings and reported that those programs have successfully identified and educated people at risk (Ferdinand et al., 2012).

However, we found that participating in CBHIs for NCDs has no significant association with the uptakes of basic vision examinations. One of the plausible explanations is that the eye care infrastructure and human resource availability vary significantly across the Primary Health Care Centres (*Puskesmas*). A qualitative study in West Java found that the availability of necessary equipment and resources are among the barriers to getting eye care (Marella et al., 2019). More than half (54%) of ophthalmologists in Indonesia are concentrated on Java island, leaving the rest of the islands with low access to eye care (Kusnawan et al., 2015). This condition may lead to unequal eye health care services in different areas of the country. Primary eye care in Indonesia will thus require a substantial investment, especially in outer Java island and rural areas in the coming years. Another explanation is that the health providers, including eye care, in Indonesia are mainly private providers. The proportion of government contribution to the total health expenditure was only 37.8%, meaning that most of the health spending has been financed by the private sector (Mahendradhata et al., 2017; Marella et al., 2019). This was supported by the study in West Java, which further revealed that direct costs are among the barriers to getting eye care (Wray et al., 2022).

We further found a negative relationship between CBHIs participation and prostate cancer screening among the male respondents. These findings contrast with prior research in high-income (Owens, 2015) and LMICs countries that show CBHIs participation improves the participants' prostate cancer screening (Zare et al., 2016). An RCT, using data from 210 men aged 50–70 in Iran, found that CBHI was associated with a higher probability of having prostate cancer screening (Zare et al., 2016). The study designed health education programs based on the health belief model to improve the respondents’ knowledge level, positively affecting perceived susceptibility and severity, and considering the perceived barriers, benefits and health motivations. The negative association between CBHIs participation and prostate cancer screening in this study may be due to the low proportion of men (1.7%) participated in CBHIs compared to women (4.5%). Prior qualitative study in Indonesia revealed that *Posbindu PTM* and *Posyandu Lansia* services relied on woman volunteers, and they attracted more woman than man participants (Pratono & Maharani, 2018). Women volunteers may also feel uncomfortable discussing prostate cancer with the male participants.

Some predisposing, enabling and need factors that have policy implications are identified. Firstly, we found that the prevalence of NCD increases with age, and older people thus are more likely to be aware of their health or develop NCDs symptoms (Chang et al., 2019). However, developing countries bear a greater burden of NCD mortality than high-income countries (L. Allen et al., 2017a, Allen et al., 2017b). The rates of premature deaths in Indonesia were significantly higher for cerebrovascular disease, diabetes, and asthma (Schröders et al., 2017). The CBHIs thus need programs to attract younger people to have NCDs screening to prevent those premature deaths. Secondly, education and wealth are associated with higher odds of having NCDs screening in this study. This finding was consistent with previous studies from different countries (Gonfa et al., 2021).

Knowledge is one of the most important driving factors in behavioural change (Li et al., 2016). Women with formal education were nearly two times more likely to have a good awareness of NCDs screening during the preconception period than those without formal education (Gonfa et al., 2021). Attending education may give the respondents the chance to gain information directly from their education programs or indirectly from discussions with others. Consistent with previous studies (Keetile et al., 2021; Negi et al., 2022; Subramanian et al., 2018), we showed that the less wealthy participants were less likely to report having NCDs screening compared to wealthier participants. This reemphasises the notion that individuals with the financial means overcome barriers to accessing care compared to those who are poor. One of the barriers to attending *Posyandu Lansia* is access (Pratono & Maharani, 2018). Older people found it difficult to visit *Posyandu Lansia* as they did not have affordable transportation. Furthermore, although *Posyandu Lansia* provided free services, some older people stated that they could not afford the cost of social events.

Our study and findings have some inherent limitations. Firstly, the information on the duration, mode and frequency of the CBHIs’ participation was not available in the data source (IFLS). This absence of data limits our analysis to understand better whether different exposure of the programs would affect the NCDs screening uptakes. The second limitation is that this study used self-reported information on NCDs screening uptakes, which may have led to potential bias due to recall issues or subjectivity in reporting (Chan, 2009, pp. 309–336). Finally, the proportion of respondents attending CBHIs was quite low (3%), which may lead to biased estimates. We tried to address the issue by performing firth logistic regressions as suggested by King and Zeng (2001). Nevertheless, in most literature in statistics, rare events have proven difficult to explain and predict (King and Zeng, 2001).

In spite of those limitations, the present study makes several noteworthy contributions to the literature on CBHIs and NCD prevention as well as assisting policymakers in determining the role of CBHIs in improving NCDs screening uptakes in Indonesia. First, this study has established evidence of the benefits of CBHIs for health care and health using a nationally-representative data in Indonesia. While most previous studies have reported the benefits of CBHIs for access to family planning and child health (Yip et al., 2007), and tackling communicable diseases, we show evidence of a link between participation in CBHIs and a higher likelihood of having NCDs screening (Ekman, 2004; Fauveau et al., 1991). Second, participation in CBHIs is associated with higher odds of having NCDs screening, except for prostate cancer screening and basic eye health examination. For policymakers, these findings suggest the need for additional strategies and services for improving access to both services and tailored educational programs to increase the uptakes of those screening tests.

## 5 Conclusion

This study showed that participation in CBHIs was associated with a higher likelihood of NCD screenings in Indonesia. These results highlighted the important role of the community in preventing NCDs. However, these results should be cautiously interpreted as the proportion of respondents attending the CBHIs was quite low (3%), and most of the attendants were older people and women. Thus, policymakers and health practitioners must design programs attractive to the younger population and men as they have a higher risk of premature deaths due to NCDs. Furthermore, our study provides a unique insight into that NCDs’ prevention should provide an “enabling” environment, including better healthcare access and health promotion, which assists people in choosing and maintaining healthy lifestyles.

## Ethical statement

The research has been performed in accordance with the Declaration of Helsinki. The IFLS surveys and their procedures were reviewed and approved by the following ethics committees: IRBs (Institutional Review Boards) in the United States (at RAND) and in Indonesia at the Universitas Gadjah Mada (UGM) for IFLS5. Further information about ethical approval is available on RAND website https://www.rand.org/labor/FLS/IFLS.html. Informed consent was obtained from all participants.

## Authors’ contributions

SS and AM conceptualised the study. SS prepared the data used for the analysis. SS performed the analysis with critical feedback from AM. All authors interpreted the results of the analysis. SS wrote the first draft. All authors reviewed and edited the draft and agreed on the final version of the manuscript.

## Consent for publication

Not applicable.

## Availability of data and materials

The dataset is publicly available at the RAND Corporation's website (http://www.rand.org/labor/FLS/IFLS/ifls5.html).

## Declaration of competing interest

The authors declare that they have no competing interests.

## Data Availability

The dataset is publicly available at the RAND Corporation's website (http://www.rand.org/labor/FLS/IFLS/ifls5.html).
