# Supplementary material for: Participation in community-based healthcare interventions and non-communicable diseases early detection of general population in Indonesia
Source: SSM Popul Health. 2022 Sep 22;19:101236. doi: 10.1016/j.ssmph.2022.101236 (PMC9513697; doi:10.1016/j.ssmph.2022.101236)
Supplement: Multimedia component 1 [file mmc1.docx]

**Supplementary file 1**

| **Variables** | **Question number in IFLS** | **Coding** |
| --- | --- | --- |
| Participate in CBHI | Book 3b_pm2 pm3type pm16_Ji | No = 0 |
|  |  | Yes = 1 |
| Sex | Book 3a_cov sex | Male = 0 |
|  |  | Female |
| Age group | Book 3a_cov age | 15-30 = 0 |
|  |  | 31-45 = 1 |
|  |  | 46-65 = 2 |
|  |  | 65-99 = 3 |
| Marriage status | Book 3a_cov | Unmarried = 0 |
|  |  | Married = 1 |
|  |  | Separated = 2 |
|  |  | Divorced = 3 |
|  |  | Widowed = 4 |
|  |  | Cohabitate = 5 |
| Education | Book 3a_dl1 dl06 dl07 | No schooling = 0 |
|  |  | Elementary = 1 |
|  |  | JSE = 2 |
|  |  | High school = 3 |
|  |  | University = 4 |
| Monthly household expenditure (IDR) | Book pce14nom pce |  |
| Religion |  | Non-Muslim = 0 |
|  |  | Muslim = 1 |
| Ethnicity | Book 3a_dl1 dl10e | Java = 1 |
|  |  | Non-Java = 0 |
| Resident living | Book bk_sc1 sc05 | Rural = 0 |
|  |  | Urban = 1 |
| Smoking status | Book 3b_km km01a | No = 0 |
|  |  | Yes = 1 |
| Physical activity | Book 3b kktype kk02o | Sedentary = 1 |
|  |  | Lightly activity = 2 |
|  |  | Moderate activity = 3 |
|  |  | Vigorous activity = 4 |
| Sedentary | Book 3b kktype kk02o | No = 0 |
|  |  | Yes = 1 |
| Openness | Book 3b psntype psn01 | <4 = 0 |
|  |  | ≥4 = 1 |
| Conscientiousness | Book 3b psntype psn01 | <4 = 0 |
|  |  | ≥4 = 1 |
| Extroversion | Book 3b psntype psn01 | <4 = 0 |
|  |  | ≥4 = 1 |
| Agreeableness | Book 3b psntype psn01 | <4 = 0 |
|  |  | ≥4 = 1 |
| Neuroticism | Book 3b psntype psn01 | <4 = 0 |
|  |  | ≥4 = 1 |
| *Enabling factors* |  |  |
| Travel time | Book 3b rj12 | <10 minutes = 1 |
|  |  | ≥10 minutes = 0 |
| Health insurance ownership | Book 3b_ak2 ak06 | No = 0 |
|  |  | Yes = 1 |
| *Need factors* |  |  |
| Parent died from CVD | Book 3b ba06e_A | No = 0 |
|  |  | Yes = 1 |
| Parent died from diabetes | Book 3b ba06e_D | No = 0 |
|  |  | Yes = 1 |
| Parent died from cancer | Book 3b ba06e_E | No = 0 |
|  |  | Yes = 1 |
| Number of comorbidities | Book 3b cdtype cd05 | <1 = 0 |
|  |  | ≥2 = 1 |
| CESD | Book 3b kp02 | <8 = 0 |
|  |  | ≥8 = 1 |
| BMI | Book bus_cov us06 us04 | <25 kg/m2 = 0 |
|  |  | ≥25 kg/m2 = 1 |
| Menopausal status | Book 4_kw2 kw23d | Premenopausal = 0 |
|  |  | Postmenopausal = 1 |
| Age at menarche | Book 4_kw2 kw23b | <14 = 0 |
|  |  | ≥14 = 1 |
| Blood pressure test | Book 3b rj24type rj24a_A | Never = 0 |
|  |  | Irregular = 1 |
|  |  | Regular = 2 |
| Cholesterol test | Book 3b rj24type rj24a_B | Never = 0 |
|  |  | Irregular = 1 |
|  |  | Regular = 2 |
| Blood glucose test | Book 3b rj24type rj24a_C | Never = 0 |
|  |  | Irregular = 1 |
|  |  | Regular = 2 |
| Electrocardiogram | Book 3b rj24type rj24a_D | Never = 0 |
|  |  | Irregular = 1 |
|  |  | Regular |
| Basic eye and vision exam | Book 3b rj24type rj24a_E | Never = 0 |
|  |  | Irregular = 1 |
|  |  | Regular = 2 |
| Basic dental exam | Book 3b rj24type rj24a_F | Never = 0 |
|  |  | Irregular = 1 |
|  |  | Regular = 2 |
| Prostate cancer test | Book 3b rj24type rj24a_G | Never = 0 |
|  |  | Irregular = 1 |
|  |  | Regular = 2 |
| Pap smear | Book 3b rj24type rj27x | Never = 0 |
|  |  | Ever = 1 |
| Breast self-exam | Book 3b rj24type rj29x | Never = 0 |
|  |  | Ever = 1 |
